# Sweetness profiles of glycosylated rebaudioside A and its binary mixtures with allulose and maltitol

**DOI:** 10.1007/s10068-020-00873-w

**Published:** 2021-02-16

**Authors:** Jinsil Jung, SooHyun Kim, Sunghee Park, Jae-Hee Hong

**Affiliations:** 1 Department of Food and Nutrition, Seoul National University, Seoul, Republic of Korea; 2 CJ CheilJedang Research Institute, Suwon, 16495 Republic of Korea; 3 Research Institute of Human Ecology, Seoul National University, Seoul, Republic of Korea

**Keywords:** Sweetener, Glycosylation, Rebaudioside A, Relative sweetness, Sensory profile

## Abstract

Rebaudioside A is a promising natural alternative sweetener but they produce increased bitterness, astringency, and unpleasant aftertastes. Glycosylation and blending with different sweeteners are known to improve the sensory characteristics of rebaudioside A. The present study was conducted to identify the relative sweetness and sensory profile of glycosyl rebaudioside A (g-reb A). The relative sweetness of g-reb A compared to 5% sucrose was determined using the two-alternative forced choice method. The sensory profiles of g-reb A and its mixtures with allulose and maltitol (1:1 ratio) were compared to those of rebaudioside A, rebaudioside D, rebaudioside M, sucralose, allulose, maltitol, and sucrose using descriptive analysis conducted by eight trained panelists. The relative sweetness of g-reb A was 155, which was lower than that of rebaudioside A. In addition, the bitter taste and aftertaste, astringency, and sweet onset of g-reb A were decreased compared to those of rebaudioside A.

## Introduction

Excessive intake of sugar can lead to obesity, type 2 diabetes, and cardiovascular disease; thus, there is a growing consumer demand for low-calorie intense sweeteners as alternatives (Malik et al., 2010), particularly natural sweeteners that do not contain synthetic or chemical ingredients with potential adverse health effects (Hellfritsch et al., 2012). Steviol glycosides are some of the most widely used natural sweeteners (Nabors, 2011).

Steviol glycosides are derived from the leaves of *Stevia rebaudiana* (Bertoni) Bertoni (Asteraceae) (candyleaf); they contain a complex mixture of diterpene glycosides including stevioside, rebaudioside A-E, steviobioside, and dulcoside A (Nabors, 2011). Although all steviol glycosides have the same aglycone, steviol (13-hydroxy-ent-kaur-16-en-19-oic acid), their sweetness intensities and profiles can differ (Kinghorn, 2001). Currently, 43 of the steviol glycoside species naturally present in *S. rebaudiana* have been identified (Gerwig et al., 2016). In general, stevia leaves contain 7–15% steviol glycosides, of which stevioside accounts for 4.0–8.5% and rebaudioside A accounts for 1.5–5.0%, while rebaudioside C and dulcoside A account for 0.1–2.5% and 0.1–1.0%, respectively (Nabors, 2011).

Stevioside and rebaudioside A are 150–250-fold and 200–300-fold sweeter than sugar, respectively (Nabors, 2011). However, these sweeteners impart a bitter taste, metallic or licorice flavors, and their sweetness is expressed slower than that of sugar (Kim et al., 2016). The bitterness of these sweeteners is due to binding with the bitter receptors hTAS2R4 and hTAS2R14 (Hellfritsch et al., 2012). Their sweetness profiles make industrial application of these sweeteners difficult because their use can change the sensory characteristics of the final product and potentially decrease consumer acceptability.

Some steviol glycosides, such as rebaudioside A, have weaker off-flavors than that of stevioside (Prakash et al., 2008). Therefore, better-tasting steviol glycosides are under investigation with the aim of developing sweeteners with sensory profiles closer to that of sucrose. Besides rebaudioside A, which is now widely applied in the industry, rebaudioside D and M (both less bitter than rebaudioside A) have been the subject of such research. However, rebaudioside D and M have been found to exhibit slight but nevertheless detectable bitterness, astringency, and licorice aftertaste (Allen et al., 2013; Prakash et al., 2014).

As a method for improving the sensory characteristics of steviol glycosides, glycosylation has shown promise. For example, transglucosylation using α-amylase reduces the bitter aftertaste of stevioside and increases its sweetness intensity (Ye et al., 2013). In addition, transglucosylation with glucansucrase from *Lactobacillus reuteri* 180 can reduce the bitterness, astringency, licorice off-flavor, and sweetness persistence in stevioside or rebaudioside A (Devlamynck et al., 2019; Te Poele et al., 2018).

As an alternative to glycosylation, blending different sweeteners has been used as a strategy for increasing sweetness potency and reducing off-flavor. For instance, the sweetness intensities of cyclamate–sucrose, glucose–sucralose, and lactose–saccharin binary mixtures were significantly increased compared to those of the single sweeteners used in each mixture (Kersch-Counet et al. 2014). Furthermore, Prakash et al. (2008) suggested that blending high concentrations of rebaudioside A with other sweeteners reduced its bitterness and licorice flavor to undetectable levels (Prakash et al. 2008).

In industrial production, intense sweeteners are commonly blended with bulk sweeteners such as carbohydrate sugars or sugar alcohols (Kim et al. 2003; Portmann and Kilcast, 1998). Recently, sugars and sugar alcohols with health-promoting effects have been considered as candidates for sweetener blends. On example, allulose (D-psicose) is a novel low-calorie rare sugar with a sweetness 70% that of sugar (Zhang et al., 2016) that has been shown to suppress postprandial blood glucose levels and produce antihyperlipidemic and antiinflammatory effects (Matsuo et al., 2001; Moller and Berger, 2003). When used alone, allulose is less sweet and bitterer than sucrose, but a 1:1 mixture of allulose–sucrose exhibits a similar profile to sucrose (Tan et al., 2019). Moreover, Woodyer et al. (2017) reported that an allulose–fructose–sucralose mixture was sweeter than any individual sweetener in the mixture. Another low-calorie sweetener, maltitol (2.1–2.4 kcal/g), helps prevent caries, suppress postprandial blood glucose, and has a low-glycemic index; it is 0.9-fold sweeter than sucrose but has a similar sweetness profile (EFSA, 2011). Using maltitol with cyclamate and acesulfame K has been shown to increase the sweetness intensity and mask the bitter, licorice, burnt-sugar, metallic, and menthol-like off-flavors of intense sweeteners; thus, mixing maltitol in this way results in a clean, sweet taste and desirable mouthfeel (Portmann and Kilcast, 1998).

Considering these various methods, the present study was conducted to determine the relative sweetness and sensory profile of glycosyl rebaudioside A (G-reb A), a novel and natural intense sweetener. In addition, G-reb A was mixed with two bulk sweeteners, allulose and maltitol, to assess potential synergistic increases in sweetness intensity and improvements in taste qualities.

## Materials and methods

### Ethics statement

All research protocols were reviewed and approved by the institutional review board of the Seoul National University (IRB No. 1908/003-002). Informed consent was obtained from all participants for inclusion in the study.

## Materials

G-reb A with 99% purity was provided by CJ CheilJedang Research Institute (Suwon, Korea). Unlike conventional enzymatically-modified stevia, in which α-1,4 glucose is randomly bound, G-reb A used here is a novel product in which α-1,6 glucose is site-specifically bound at C19 of the steviol glycoside. The sensory properties of g-reb A were investigated in comparison with other rebaudioside sweeteners, including rebaudioside A (Reb A), rebaudioside D (Reb D), and rebaudioside M (Reb M) (both Shandong Haigen Biotechnology Co., Qufu, China). Sucralose (Sucral; Tate & Lyle Ingredients Americas LLC, Decatur, USA), which is known to have sensory characteristics close to those of sucrose, was used for comparison. Sucrose (Suc; white sugar; CJ CheilJedang Co., Incheon, Korea) was used as the control. Allulose (Al; CJ CheilJedang Co., Gyeonggi-do, Korea) and maltitol (Mal; Roquette China Co., Lianyungang, China) were used for blending to assess the effects on the sensory properties of G-reb A. All samples except sucrose and sucralose were provided by CJ CheilJedang Research Institute (Suwon, Korea).

### Analysis of relative sweetness using the two-alternative forced choice method

#### Panel

Panelists who had no problems with the intake of sugars or alternative sweeteners were recruited by posting flyers at Seoul National University (Seoul, Korea) and in the surrounding local community. Those who might be or were pregnant or those who had a potential health risk (e.g. diabetes or allergy) associated with sweetener consumption were excluded. A total of 138 panelists (66 men and 72 women aged 18–45 years) were recruited for the two-alternative forced choice (2-AFC) test, while one type of sweetener was evaluated by 54–69 panelists.

#### Sample preparation

The five concentration levels of each sweetener were set based on a literature review and preliminary experiments (Table 1). One day before the test, each sweetener was dissolved in filtered water(everpure H-300, Pentair Residential Filteration, LLC., Brookfield, USA) at room temperature (18 °C ± 2 °C) using a magnetic stirrer (MS300HS, Misung Scientific Co., Ltd., Yangju, Korea) for 10 min at 1100 rpm, and then stored in a refrigerator (3 °C ± 1 °C) until further use. Before their evaluation, samples were equilibrated at room temperature (22 °C ± 2 °C) for 3 h, and then 10 mL of each sample was placed into a white plastic cup (diameter 7 cm, height 3 cm; Taesanpack Co., Gyeonggido, Korea) labeled with a three-digit random number. Through a preliminary experiment, it was confirmed that sensory characteristics did not change during storage and equilibration periods. Warm water (46 °C ± 2 °C) and filtered water at room temperature (22 °C ± 2 °C) were provided to the panelists during testing for palate cleansing.

**Table 1:** Concentrations of the sweetener samples used in the two-alternative forced choice test

Sample	Sample abbreviation	Concentration (w/v^1^, %)
Glycosyl rebaudioside A	G-reb A	0.01, 0.02, 0.03, 0.04, 0.05
Rebaudioside A	Reb A	0.01, 0.02, 0.03, 0.04, 0.05
Rebaudioside D	Reb D	0.005, 0.011, 0.017, 0.023, 0.029
Rebaudioside M	Reb M	0.005, 0.011, 0.017, 0.023, 0.029
Sucralose	Sucral	0.004, 0.006, 0.008, 0.01, 0.012
Allulose	Al	6, 7.5, 9, 10.5, 12
Maltitol	Mal	5.5, 6.5, 7.5, 8.5, 9.5

^1^Weight per volume

#### Evaluation procedure

Using the 2-AFC test protocol established by Kim et al. (2015), the sweetness of each sweetener was determined relative to a 5% sucrose aqueous solution. In one session, one type of sweetener was evaluated by testing five pairs consisting of a sweetener solution at various concentrations and the 5% sucrose solution. Panelists were randomly assigned to a total of four sessions. The order of sessions and presentation of the five pairs within a session were decided following a William Latin square design. The serving order of samples within a pair was randomized and counter-balanced.

Panelists were asked to assess overall sweetness for 10 s, first by swirling the entire 10 mL of the sample in the oral cavity for 5 s and then by perceiving sweetness for 5 s after expectorating the sample. They were asked to choose the sweeter sample in a given sample pair. Panelists were instructed to rinse their mouth first with warm water and then with filtered water and take a break for 1 min between the samples and to take a 5 min break between sessions to minimize desensitization and fatigue. They were also asked to refrain from consuming food, drinking liquids other than water, and using scented personal care products and perfumes 1 h prior to the evaluation.

From the results, concentration–response curves (C–R curves) were constructed by plotting the ratio of responses by which a sample was sweeter than the 5% sucrose solution against the concentration levels. The concentration that produced 50% of such responses was calculated from the regression equation of the C-R curve. Relative sweetness values were determined by dividing the concentration of sucrose (5%) by the sweetener concentration that yielded 50% of the responses.

### Characterization of the sensory attributes of G-reb A using descriptive analysis

#### Sample preparation

Samples were prepared to have a sweetness equivalent to that of the 5% sucrose solution based on results of the 2-AFC tests (Table 2). Sweetener blends were prepared by mixing G-reb A solution with Mal or Al solutions at a 1:1 ratio. Samples were prepared using the same method as for the 2-AFC test described above. A 40 mL aliquot of each sample was presented in a white disposable plastic cup labeled with a three-digit random number.

**Table 2:** The sample concentrations used in the descriptive analysis

Sample^1^	Concentration (w/v^2^)
Suc	5.0000%
G-reb A	0.0322%
Reb A	0.0213%
Reb D	0.0209%
Reb M	0.0190%
Sucral	0.0084%
Al	9.3877%
Mal	7.6388%
G-reb A + Al	1:1 mixture of 0.0322% G-reb A and 9.3877% Al
G-reb A + Mal	1:1 mixture of 0.0322% G-reb A and 7.6388% Mal

^1^See Table 1 for sample abbreviations

^2^Weight per volume

#### Evaluation procedure

The sensory profiles of the sweeteners were determined using the generic descriptive analysis procedure (Lawless & Heymann, 2010). Eight panelists (three men and five women aged 19–31) who had no problems with the intake of sucrose or alternative sweeteners were recruited. At the first training session, panelists were introduced to the purpose of the study and educated in the principles of the descriptive analysis protocol. Training was conducted to establish a consensus in the lexicon, tasting and rinsing protocols, scale use, and reference samples (Table 3). Practice tests were conducted in quadruplicate to assess the panelists’ performance. Analysis of variance (ANOVA) was conducted on the practice test data to test significance of the panel effect or interaction effects between panel and other factors, in order to check if the panelists could rate samples consistently across repetitions as well as with the group results. Additional training was provided to those who had poor reproducibility or different rating patterns from the group result. Panel training was performed 2–3 times a week for 3 months. Each training session lasted 1.5 h.

**Table 3:** Definitions and reference materials for the descriptive attributes of sweetener samples

Attributes	Definition	Reference materials	Scale value (0–15)
Taste			
Sweetness	Fundamental taste sensation of which sucrose is typical	2% (w/v^1^) sucrose (CJ CheilJedang Co., Ltd., Korea), 5% and 10% sucrose in water	3.4, 7.5, and 13.5
Bitterness	Fundamental taste sensation of which caffeine and quinine is typical	0.03% (w/v) caffeine (Sigma-Aldrich, St. Louis, MO, USA) in water	11.3
Sourness	Fundamental taste sensation of which citric flavor is typical	0.03% (w/v) citric acid (RZBC Co., Ltd., China) in water	13.5
Flavor			
Honey flavor	Honey flavor typically found in Acacia honey	2% (w/v) acacia honey (Seoraksanmilbongwon, Korea) in water	11.3
Trigeminal senses			
Astringency	The feeling which shrivels the tongue associated with aluminum potassium sulfate	0.1% (w/v) aluminum potassium sulfate (Daejung Chemicals & Metals Co., Gyeonggi-do, Korea.) in water	15
Acridness	Sharp, irritating, or biting sensation on the tongue	10% (w/v) erythritol (Zibo Zhongshi Green Biotech Co., Ltd., China) in water	7.5
Mouthfeel			
Body	Rate of sample flow on the tongue	1.5 g potato starch (potato starch, Sunginfood, Gyeounggi-do, Korea) and 500 mL water (boiled on a low heat with stirring for ~ 5 min)	N/A^2^
Temporal aspect			
Sweet aftertaste	Sweet taste for 5 s after expectoration	0.05% (w/v) aspartame (The Nutrasweet Co., USA) in water	13.75
Bitter aftertaste	Bitter taste for 5 s after expectoration	0.03% (w/v) caffeine (Sigma-Aldrich, St. Louis, MO, USA) in water	15
Onset of sweetness	The time at which maximum sweetness was first perceived	Within 1 s Within 3–4 s	1.25 13.75

^1^Weight per volume

^2^N/A: the reference standard of the sensory attribute did not have a specific scale value because the reference sample was only used for the panelists’ concept alignment

The intensity of sensory characteristics was rated on a 15 cm line scale (1.25 cm = very weak/very fast; 13.75 cm = very strong/very slow). The tests were repeated four times. Samples were presented in a monadic sequence according to the William Latin square design. The panelists thoroughly rinsed their mouth with warm and filtered water and rested for 3 min between samples and for 5 min after evaluating four samples to minimize sensory and mental fatigue. They were instructed to expectorate samples to minimize health problems that may arise from intake of alternative sweeteners.

### Data analysis

An analysis of variance (ANOVA) was conducted to test for significant differences in the intensities of sensory attributes among samples. The ANOVA model included sample, panel, repetitions, and their two-way interactions. Tukey’s HSD test (*p* < 0.05) was conducted as a post hoc analysis. Principal components analysis (PCA) was performed using the mean intensities of sensory attributes to identify the multivariate relationships among samples and sensory attributes. Data was centered by panelists to remove a scale effect for PCA. ANOVA and post hoc testing were performed using SPSS (version 25.0; IBM Inc., Armonk, NY, USA), whereas PCA was conducted using the FactoMineR package (Lê et al., 2008) in R (version 3.3.2; R Foundation for Statistical Computing, Vienna, Austria).

## Results and discussion

### Relative sweetness of sweeteners

The relative sweetness, regression equation, and regression coefficient (R^2^) were calculated from the C-R curve of each sweetener (Table 4). The R^2^ values ranged from 0.92 to 0.99, indicating that the regression model explained the data well. The relative sweetness values of Reb A, Reb D, Reb M, Sucral, Mal, and Al were similar to those reported in previous studies (Table 4; Gwak et al., 2012; Ko et al., 2020; Nabors, 2011; Prakash et al., 2014; Schiffman et al., 1995).

**Table 4:** Relative sweetness of seven sweeteners calculated from concentration–response curves

Sample^1^	Regression equation	R^2^	Relative sweetness determined using 2-AFC^2^	Relative sweetness from literature
G-reb A	y = 22.174x − 0.213	0.9459	155	–
Reb A	y = 0.4791ln(x) + 2.3444	0.9688	235	227.3 (Gwak et al., 2012) 200-300 (Nabors et al., 2011)
Reb D	y = 33.871x − 0.2081	0.9747	239	221 (Prakash et al., 2014)
Reb M	y = 34.906x − 0.1632	0.94	263	250 (Prakash et al., 2014)
Sucral	y = 105.56x − 0.3852	0.9186	596	600 (Nabors, 2011)
Al	y = 0.1264x − 0.6866	0.9472	0.53	0.6 (Ko et al., 2020)
Mal	y = 0.1877x − 0.9338	0.9891	0.65	0.67 (Gwak et al., 2012) 0.72 (Schiffman et al., 1995)

^1^See Table 1 for sample abbreviations

^2^The protocol developed by Kim et al. (2015) was applied for construction of dose–response curve using 2-AFC test

The relative sweetness of G-reb A was 155, which was lower than that of unglycosylated rebaudioside A (Table 4). The effect of glycosylation on relative sweetness varies across previous studies. For example, Ko et al. (2016) observed an increase in the relative sweetness of steviosides as a result of glycosylation, whereas Te Poele et al. (2018) and Devlamynck et al. (2019) found that glycosylation decreased the sweetness of steviosides. It is assumed that the effect of glycosylation is dependent on the location or amount of glycosylation in given molecules. Gerwig et al. (2016) found that an increase in the number of β-glucosyl residues caused the sweetness or bitterness of steviol. Devlamynck et al. (2019) reported that the bitter taste and aftertaste of stevioside were decreased after glycosylation, and that multi-alpha-glycosylation at C-19 and C-13 reduced the sweetness of steviosides while mono-alpha-glycosylation at beta-glucose residue C-19 did not. G-reb A used in the present study has 1–4 glucose molecules bound at C-19 of steviol glycoside with an α-1,6-glycosydic linkage. Therefore, glycosylation of G-reb A seems to improve its sweetness quality rather than increase its sweetness intensity.

The relative sweetness of most samples was fitted well to the linear regression model, but that of Reb A was better explained by logarithmic regression (R^2^: 0.97; Table 4). The studies of Choi and Chung (2014) and Ko et al. (2020) also indicated that logarithmic regression models can better explain the relative sweetness of some intense sweeteners. In some cases, the strong bitterness and off-flavors of intense sweeteners at high concentrations can mask sweetness, thereby flattening the C-R curve in the high concentration range (Choi and Chung, 2014; Kim et al., 2015; Kim et al., 2016). In the present study, the strong bitter taste, aftertaste, and astringency of Reb A (Table 5) suppressed its sweet taste at high concentrations, which caused a logarithmic C-R curve. However, the relative sweetness of Al, which was found to have a strong bitter taste (Table 5), was better fitted to the linear model. In a previous study, DuBois (2016) reported that a linear model explained the concentration-dependent increase in the perceived sweetness intensities of carbohydrate sweeteners, whereas a curvilinear model better explained the sweetness responses of intense sweeteners. DuBois (2016) also suggested the molecular mechanism of sweetness to be as follows. Only part of the binding sites of sweet taste receptors T1R2/T1R3 remains active; thus, carbohydrate sweeteners activate these receptors not only through direct binding but also through a constitutional active modulator reaction, which activates all binding sites, whereas intense sweeteners likely activate the receptors through direct binding only.

**Table 5:** Mean intensities of the descriptive sensory attributes elicited by sucrose and nine sweeteners

Sample^1^	Suc	G-reb A	Reb A	Reb D	Reb M	Sucral	Al	Mal	G-reb A + Al	G-reb A + Mal
Sweetness	7.5^abcd2^	7.7 ^cde^	6.9^ab^	7.1^abc^	7.6^abcd^	6.8^a^	8.4^e^	8.0^de^	7.6^bcde^	8.1^de^
	(0.3)^3^	(0.7)	(1.3)	(1.2)	(1.2)	(1.2)	(1.4)	(1.1)	(0.9)	(1.0)
Bitterness	0.8^a^	2.7^cd^	5.3^e^	2.6^c^	3.5^d^	1.9^bc^	4.8^e^	1.5^ab^	4.7^e^	1.6^ab^
	(1.1)	(2.3)	(1.6)	(2.4)	(2.5)	(1.7)	(2.0)	(1.4)	(2.1)	(2.0)
Sourness	1.2^a^	2.7^bc^	3.0^bcd^	2.7^bc^	3.2 ^cd^	2.5^bc^	5.8^e^	1.9^ab^	2.6^bc^	4.1^d^
	(1.5)	(2.0)	(2.2)	(2.2)	(2.4)	(2.4)	(1.9)	(1.9)	(1.9)	(2.4)
Honey flavor	2.0^ab^	2.7^b^	1.8^ab^	1.5^a^	2.0^ab^	1.6^a^	5.6^c^	5.6^c^	2.3^ab^	2.8^b^
	(1.5)	(2.3)	(2.0)	(1.6)	(1.9)	(1.5)	(1.8)	(1.7)	(1.7)	(2.0)
Astringency	1.0^a^	3.1^c^	5.5^d^	3.1^c^	4.9^d^	2.6^bc^	5.5^d^	2.0^b^	4.9^d^	2.0^ab^
	(0.8)	(2.1)	(1.4)	(2.2)	(2.0)	(2.0)	(1.7)	(1.8)	(1.8)	(2.1)
Acridness	0.9^a^	2.3^bc^	2.9^bcd^	2.0^b^	2.6^bcd^	2.1^b^	6.0^e^	3.5^d^	3.2 ^cd^	2.6^bcd^
	(1.0)	(1.7)	(2.2)	(1.8)	(2.1)	(1.9)	(1.8)	(2.3)	(1.8)	(2.1)
Body	3.6^ab^	3.5^ab^	3.7^ab^	3.7^ab^	3.5^ab^	3.1^a^	4.0^b^	5.3^c^	4.0^b^	5.3^c^
	(2.6)	(2.5)	(2.6)	(2.5)	(2.2)	(2.5)	(2.5)	(2.3)	(2.5)	(2.3)
Sweet aftertaste	3.9^a^	7.1^d^	4.3^ab^	4.2^ab^	5.1^bc^	4.1^ab^	5.4^c^	5.2^bc^	4.7^abc^	7.5^d^
	(2.4)	(2.0)	(2.7)	(2.6)	(2.5)	(2.2)	(2.7)	(2.7)	(2.1)	(2.1)
Bitter aftertaste	0.8^a^	2.4 ^cd^	5.6^e^	2.3 ^cd^	3.2^d^	1.9^abc^	4.9^e^	1.2^ab^	2.3^bcd^	1.7^abc^
	(1.0)	(2.1)	(1.9)	(2.2)	(2.6)	(2.2)	(2.1)	(1.2)	(2.2)	(1.9)
Onset of sweetness	3.2^a^	4.2^ab^	6.4^c^	4.4^b^	4.3^ab^	6.2^c^	4.7^b^	6.0^c^	4.6^b^	3.7^ab^
	(2.0)	(1.9)	(1.9)	(2.4)	(2.0)	(2.4)	(1.8)	(2.0)	(2.1)	(1.7)

^1^See Table 1 for sample abbreviations

^2^Different lowercase letters indicate significant differences among the sweetener samples (*p* < 0.05)

^3^Standard deviation

### Sensory characteristics of sweeteners

**Fig. 1:**
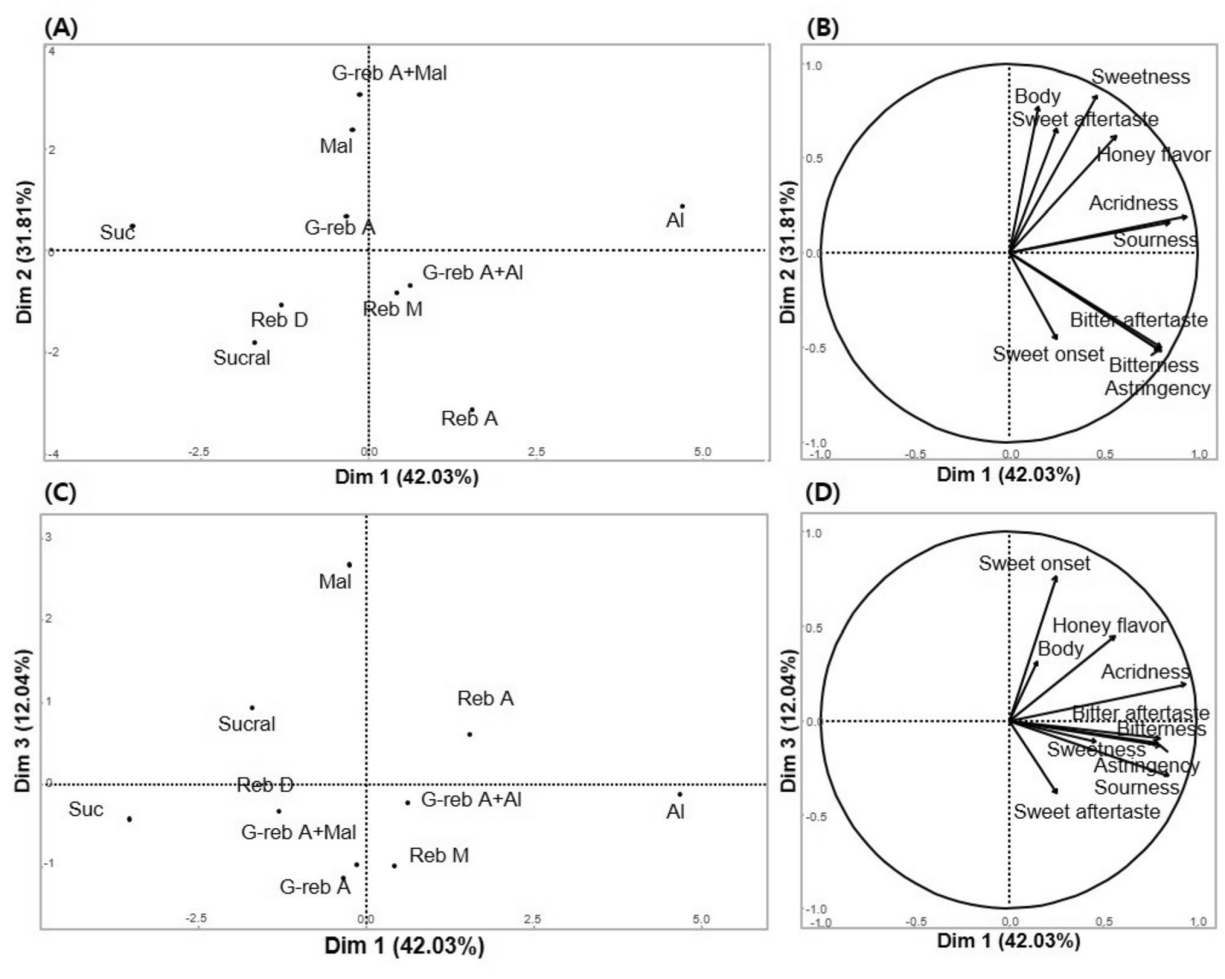
Principal component loading scores of the samples (**A**) and sensory attributes (**B**) from 10 sweeteners on Dim 1 and Dim 2, and scores of the samples (**C**) and sensory attributes (**D**) from 10 sweeteners on Dim 1 and Dim 3

There were significant differences among the samples for all sensory attributes (*p* < 0.001). The multivariate relationship between the sensory attributes and samples is shown in Fig. 1. From PCA analysis, Dim 1, Dim 2, and Dim 3 explained 42.03%, 31.81%, and 12.04% of the total variance, respectively. Dim 1 was characterized by sourness, acridness, bitter taste and aftertaste, and astringency, all of which were highly loaded in the positive direction. Dim 2 explained the increase in sweet taste and aftertaste, body, and honey flavor from its negative direction to positive direction. For Dim 3, sweet onset was highly loaded in the positive direction.

Dim 1 contrasted Al to Suc (Fig. 1A). Al had strong sourness, acridness, bitter taste and aftertaste, and astringency, whereas these characteristics were very weak in Suc (Table 5). Reb A and G-reb A + Al that were located in the positive direction of Dim 1 also had significantly stronger bitterness and astringency than the other samples (Fig. 1A, B). However G-reb A + Al had significantly weak bitter aftertaste than Reb A and Al. While these attributes were also somewhat or significantly more strongly exhibited in Reb M than in other samples, they were still weaker in Reb M than in Al, Reb A, and G-reb A + Al. Al and Reb A were the bitterest sweeteners (Table 5), which is consistent with the findings of Ko et al. (2020) who reported that Al and Reb A were significantly bitterer than sugar. The strong bitterness and acridness of Reb A were also observed by Kim et al. (2015). A previous study reported that Reb M has a less bitter taste and aftertaste, sourness, and astringency, as well as a faster onset of sweetness, than Reb A (Prakash et al., 2014). However, in the present study, there was no significant difference in sourness between Reb A and Reb M (Table 5). Reb D was significantly less bitter than Reb A (Table 5), in accordance with the findings of Allen et al. (2013).

G-reb A + Mal and Mal, which were highly loaded in the positive direction of Dim 2, had a strong sweet taste and aftertaste, body, and honey flavor. Indeed, G-reb A + Mal had a significantly stronger sweet taste and aftertaste, body, and honey flavor than the other samples, while Mal had a significantly stronger body, and sweet taste and aftertaste than other samples (Table 5). Mal is known to improve rheological properties by endowing a creamy texture (Grembecka, 2015). Al was also found to have a significantly stronger sweetness and honey flavor, as well as acridness, astringency, sourness, and bitterness than other samples. The honey flavor of Al was also previously reported by Ko et al. (2020). In the present study, G-reb A was characterized by a sweet aftertaste that was significantly stronger than that of the other samples.

It must be noted that there were significant differences in sweetness among the samples despite them being formulated to have iso-sweetness to 5% sucrose solution based on the 2-AFC test results (Table 5). This result may be attributable to differences in the tasting protocol. Gwak et al. (2012) suggested that the overall sweetness of a sweetener might be influenced by its temporal profile. In the present study, relative sweetness was determined by assessing sweetness for 5 s while holding a sample in the mouth and then for 5 s after it was expectorated. However, in the descriptive analysis, the measurement of sweetness was divided into three phases: onset, sweetness perceived when holding the sample in the mouth for 5 s, and aftertaste. In addition, aroma attributes such as honey flavor might contribute to sweetness perception through an aroma–taste interaction. Both vanilla flavor and isoamyl acetate (banana flavor) are known to increase the sweetness perception of sucrose solution (Valentin et al., 2006). Furthermore, aqueous solutions of luo han guo extract, xylo-oligosaccharides, or xylobiose each formulated to have a sweetness equivalent to 5% sucrose solution were rated as significantly sweeter than the sucrose solution by panelists during the descriptive analysis of Kim et al. (2015). These sweeteners had various sweet-note aromas such as licorice, honey, and Nurungi (toasted rice) candy.

In the PCA results, Mal, Sucral, and Reb A were highly loaded in the positive direction of Dim 3 (Fig. 1C). The sweetness of theses samples appeared more slowly than the other samples, which were highly loaded sweet onset (Fig. 1D). In contrast, the sweetness onset of G-reb A, Reb M, and G-reb A + Mal did not significantly differ from that of Suc. In a previous study, the temporal profile obtained from a time-intensity test showed that the sweetness of Reb A had a slow onset and longer persistence (Kim et al., 2015). Rebaudioside A and glycosylated stevia were also characterized as having a slow sweetness onset and strong sweetness aftertaste during descriptive analysis (Kim et al., 2016). However, the slow sweetness onset of Mal observed in the present study was not consistent with the findings of Tan et al. (2019); they assessed the temporal profile of a 10% maltitol aqueous solution using a temporal check-all-that-apply (tCATA) test and found that the onset and persistence of its sweetness were similar to those of sucrose. The inconsistency in these results might be due to differences in test protocols. In tCATA, the number of the subjects who check “sweetness” is counted over time, instead of rating sweetness intensity on a scale. Moreover, the concentrations used in the tCATA study, which were greater than 5%, might have led to faster perception of sweetness and accelerated onset of sweetness. In future studies, more elaborate protocols should be used to collect temporal measurements and identify the sweetness onset and persistence of sweeteners. In the present study, the sweetness onset of Sucral appeared slower than that of Suc, which was inconsistent with results reported by Kim et al. (2015), who tracked changes in sweetness over time using a time-intensity test in which panelists focused on only one attribute at a time, i.e., sweetness. In contrast, panelists in the present study attempted to detect the onset of sweetness while holding a sample in their mouth; this protocol might have allowed some sensory interactions, such as the suppression of sweetness by bitterness, to influence the panelists’ perception of sweetness.

Sucral, Reb D, and G-reb A were located more closely to Suc in the PCA results than the other sweeteners (Fig. 1A). Although these samples had a significantly stronger bitter taste and aftertaste, sourness, astringency, and acridness, their mean values indicate that they had “very weak” intensities (Table 5).

When the sensory profile of G-reb A was compared to those of Reb-A, D, and M, G-reb A had a significantly stronger sweet aftertaste. In addition, G-reb A was significantly less bitter and its bitterness was less persistent than Reb A, and it was also less astringent than Reb A and Reb M (Table 5). Moreover, the sweetness onset of G-reb A not only occurred significantly faster than that of Reb-A, but also showed no significant difference from that of Suc. This suggests that glycosylation improved the sensory profile of G-reb A by reducing undesirable flavor and sweetness onset. This result is in agreement with those of Te Poele et al. (2018) and Devlamynck et al. (2019) who reported a decrease in the off-flavor and bitterness of rebaudioside by glycosylation.

Mixing G-reb A with bulk sweeteners did not produce a synergistic increase in sweet taste (Table 5). However, the perceived intensities of bitterness and astringency for G-reb A + Mal were significantly weaker than for G-reb A alone as well as reb A, D, and M, and they were not significantly stronger than those of Mal. In addition, both the honey flavor and sweetness onset of this mixture were significantly weaker than those of Mal but did not differ significantly from those of G-reb A. G-reb A + Mal had a stronger sweet aftertaste than Mal, exhibiting an intensity similar to that of G-reb A. G-reb A + Mal was significantly sourer than Mal and G-reb A, respectively, implying that mixing produced a synergistic effect on sourness. In summary, mixing G-reb A with Mal suppressed the bitterness and astringency of G-reb A and increased its sourness, but it did not significantly enhance the honey flavor, sweetness persistence, and onset of sweetness, which were instead maintained at similar levels to those of Mal alone.

The sweet aftertaste of the G-reb A + Al mixture was less persistent than that of G-reb A alone, but it was not significantly different from that of Al. In addition, honey flavor, sourness, bitter aftertaste, and acridness did not change significantly by adding Al to G-reb A, suggesting that mixing the two sweeteners did not improve sweetness quality.

Schiffman et al. (1995) reported that 1:1 mixtures of Reb A and various sweeteners, including intense sweeteners such as acesulfame K, aspartame, alitame, and sodium cyclamate and bulk sweeteners such as fructose, glucose, and mannitol, exhibited additivity of sweetness rather than synergy at the 5% relative sweetness level. However, these mixtures exhibited suppression of sweetness at the 10% relative sweetness level. The results of the present study were consistent with those of Schiffman et al. (1995) at the 5% relative sweetness level; however, the present results also suggest that Mal is a promising sweetener for improving sweetness quality. Embuscado (2006) reported that sugar alcohols synergistically increase the sweetness of intense sweeteners, such as acesulfame K and cyclamate and impart “a full and well-balanced sweetness”, when mixed together. Kim et al. (2003) found that sorbitol, xylitol, and isomalt decrease the bitterness and astringency of aspartame when used in a mixture; however, adding sugar alcohols to intense sweeteners does not significantly affect the temporal profile of the sweeteners. Schiffman et al. (2007) reported that most binary mixtures typically reached their maximum sweetness intensities sooner than one sweetener component in a given mixture but later than the other component. However, synergistic decreases in the time to maximum intensity in a binary mixture have rarely been observed. This suggests that strategies other than mixing may be required to decrease the onset of sweetness. It is known that a variety of sweeteners and mixing ratios influence the intensity and quality of sweetness in sweetener mixtures (Hutteau et al. 1998; Schiffman et al. 1995); therefore, in future studies, sugar alcohols other than maltitol, such as xylitol and erythritol, should also be investigated for their effects on the sweetness perception of sweetener blends.

In conclusion, G-reb A and its mixture with maltitol were less bitter and astringent than Reb A, indicating an improved sensory profile, although a synergistic effect on sweetness was not observed. These findings suggest that glycosylation and blending with sugar alcohol are potential strategies for decreasing the undesirable characteristics of natural intense sweeteners. However, this reduction in undesirable attributes was not sufficient to produce a desirable sweetness quality, which is defined as a sensory profile close to that of sugar. In future studies, more sweeteners should be tested as components for blending with G-reb A to develop mixtures that improve the sensory profile of sweeteners and produce synergistic enhancements of sweetness.

## References

[CR1] Allen AL, McGeary JE, Hayes JE (2013). Rebaudioside A and rebaudioside D bitterness do not covary with acesulfame K bitterness or polymorphisms in TAS2R9 and TAS2R31. Chemosensory Perception.

[CR2] Choi JH, Chung SJ (2014). Optimal sensory evaluation protocol to model concentration-response curve of sweeteners. Food Research International.

[CR3] Devlamynck T, Te Poele EM, Quataert K, Gerwig GJ, Van de Walle D, Dewettinck K, Kamerling JP, Soetaert W, Dijkhuizen L (2019). Trans-α-glucosylation of stevioside by the mutant glucansucrase enzyme Gtf180-Delta N-Q1140E improves its taste profile. Food Chemistry.

[CR4] Dubois GE (2016). Molecular mechanism of sweetness sensation. Physiology & Behavior.

[CR5] EFSA Panel on Dietetic Products (2011), Nutrition and Allergies (NDA). Scientific Opinion on the substantiation of health claims related to the sugar replacers xylitol, sorbitol, mannitol, maltitol, lactitol, isomalt, erythritol, D‐tagatose, isomaltulose, sucralose and polydextrose and maintenance of tooth mineralisation by decreasing tooth demineralisation (ID 463, 464, 563, 618, 647, 1182, 1591, 2907, 2921, 4300), and reduction of post‐prandial glycaemic responses (ID 617, 619, 669, 1590, 1762, 2903, 2908, 2920) pursuant to Article 13(1) of Regulation (EC) No 1924/2006. EFSA J. 9: 2076 (2011) 10.2903/j.efsa.2011.2076 PMC13129610 42078783

[CR6] Embuscado ME (2006). Polyols. Chapter 8, pp. 153-174. In: Optimizing sweet taste in foods. Spillance WJ(ed). Woodhead Publishing Ltd, Cambridge, UK (2006)

[CR7] Gerwig GJ, Te Poele EM, Dijkhuizen L, Kamerling JP (2016). Stevia glycosides: chemical and enzymatic modifications of their carbohydrate moieties to improve the sweet-tasting quality. Advances in Carbohydrate Chemistry and Biochemistry.

[CR8] Grembecka M (2015). Sugar alcohols—their role in the modern world of sweeteners: a review. European Food Research and Technology.

[CR9] Gwak MJ, Chung SJ, Kim YJ, Lim CS (2012). Relative sweetness and sensory characteristics of bulk and intense sweeteners. Food Science and Biotechnology.

[CR10] Hellfritsch C, Brockhoff A, Stähler F, Meyerhof W, Hofmann T (2012). Human psychometric and taste receptor responses to steviol glycosides. Journal of Agricultural and Food Chemistry.

[CR11] Hutteau F, Mathlouthi M, Portmann MO, Kilcast D (1998). Physicochemical and psychophysical characteristics of binary mixtures of bulk and intense sweeteners. Food Chemistry.

[CR12] Kersch-Counet C, Asma R, Wassink AM, Schoen E, Dekkers R, Ponne C (2014). Chapter 95 - Synergistic/Suppressive Effects of Binary and Ternary Mixtures of Sweeteners in Semi-Skimmed Milk. pp. 513-517. In: Flavour Science. Ferreira V, Lopez R (eds). Academic Press, Inc., San Diego, CA, USA (2014)

[CR13] Kinghorn AD (2001). Stevia: The genus Stevia. CRC Press, London. p. 138 (2001)

[CR14] Kim Y, Lee J, Kim H, Choi SJ, Shin WS, Moon TW (2003). Sensory and physicochemical properties of selected sweetener blends containing polyols. Food Science and Biotechnology.

[CR15] Kim MJ, Yoo SH, Kim Y, Hong JH (2016). Relative sweetness and sweetness quality of phyllodulcin [(3R)-8-Hydroxy-3-(3-hydroxy-4-methoxyphenyl)-3,4-dihydro-1H-isochromen-1-one]. Food Science and Biotechnology.

[CR16] Kim MJ, Yoo SH, Jung S, Park MK, Hong JH (2015). Relative sweetness, sweetness quality, and temporal profile of xylooligosaccharides and luo han guo (Siraitia grosvenorii) extract. Food Science and Biotechnology.

[CR17] Ko WW, Kim SB, Chung SJ (2020). Effect of concentration range on the accuracy of measuring sweetness potencies of sweeteners. Food Quality and Preference.

[CR18] Ko JA, Nam SH, Park JA, Wee JA, Kim D, Lee WS, Ryu YB, Kim YM (2016). Synthesis and characterization of glucosyl stevioside using Leuconostoc dextransucrase. Food Chemistry.

[CR19] Lawless HT, Heymann H (2010). Descriptive analysis. Chapter 10. pp. 227-258. In: Sensory Evaluation of Food: Principles and Practices, 2^nd^ ed. Lawless HT, Heymann H. Springer, New York, USA (2010)

[CR20] Lê S, Josse J, Husson F (2008). FactoMineR: an R package for multivariate analysis. Journal of Statistical Software.

[CR21] Malik VS, Popkin BM, Bray GA, Després JP, Hu FB (2010). Sugar-sweetened beverages, obesity, type 2 diabetes mellitus, and cardiovascular disease risk. Circulation..

[CR22] Matsuo T, Baba Y, Hashiguchi M, Takeshita K, Izumori K, Suzuki H (2001). Dietary D -psicose, a C-3 epimer of D -fructose, suppresses the activity of hepatic lipogenic enzymes in rats. Asia Pacific Journal of Clinical Nutrition.

[CR23] Moller DE, Berger JP (2003). Role of PPARs in the regulation of obesity-related insulin sensitivity and inflammation. International Journal of Obesity.

[CR24] Nabors LO (2011). Alternative sweeteners.

[CR25] Portmann MO, Kilcast D (1998). Descriptive profiles of synergistic mixtures of bu1k and intense sweeteners. Food Quality and Preference.

[CR26] Prakash I, DuBois GE, Clos JF, Wilkens KL, Fosdick LE (2008). Development of rebiana, a natural, non-caloric sweetener. Food and Chemical Toxicology.

[CR27] Prakash I, Markosyan A, Bunders C (2014). Development of next generation stevia sweetener: rebaudioside M. Foods..

[CR28] Schiffman SS, Booth BJ, Carr BT, Losee ML, Sattely-Miller EA, Graham BG (1995). Investigation of synergism in binary mixtures of sweeteners. Brain Research Bulletin.

[CR29] Schiffman SS, Sattely-Miller EA, Bishay IE (2007). Time to maximum sweetness intensity of binary and ternary blends of sweeteners. Food Quality and Preference.

[CR30] Tan VWK, Wee MSM, Tomic O, Forde CG (2019). Temporal sweetness and side tastes profiles of 16 sweeteners using temporal check-all-that-apply (TCATA). Food Research International.

[CR31] Te Poele EM, Devlamynck T, Jäger M, Gerwig GJ, Van de Walle D, Dewettinck K, Hirsch AKH, Kamerling JP, Soetaert W, Dijkhuizen L (2018). Glucansucrase (mutant) enzymes from Lactobacillus reuteri 180 efficiently transglucosylate Stevia component rebaudioside A, resulting in a superior taste. Scientific Reports.

[CR32] Valentin D, Chrea C, Nguyen DH. Taste-odor interactions in sweet taste perception. Chapter 4. pp. 66-84. In: Optimizing sweet taste in foods. Spillane WJ (ed). Woodhead Publishing Ltd. Cambridge, UK (2006)

[CR33] Woodyer RD, Cohen JC, Bridges JR. Sweetener. U.S. US 9,635,879 B2 (2017)

[CR34] Ye F, Yang R, Hua X, Shen Q, Zhao W, Zhang W (2013). Modification of stevioside using transglucosylation activity of Bacillus amyloliquefaciens α-amylase to reduce its bitter aftertaste. LWT-Food Science and Technology.

[CR35] Zhang W, Yu S, Zhang T, Jiang B, Mu W (2016). Recent advances in d-allulose: Physiological functionalities, applications, and biological production. Trends in Food Science & Technology.

